# Perceived value threat of COVID-19 is related to anxiety symptoms

**DOI:** 10.1192/j.eurpsy.2021.808

**Published:** 2021-08-13

**Authors:** E. Fyodorova, G. Arina, M. Iosifyan

**Affiliations:** 1 Psychology, Lomonosov Moscow State University, Moscow, Russian Federation; 2 Psychology, University of St Andrews, School of Divinity, St Andrews, United Kingdom

**Keywords:** values, value threat, COVID-19, Anxiety

## Abstract

**Introduction:**

Recent studies showed that stress and anxiety increased during the Covid-19 pandemic (Bäuerle et al., 2020; Salari et al., 2020). It is important to identify factors which are related to this increase.

**Objectives:**

In present study we investigated how perceived value threat of Covid-19 is related to anxiety and depression symptoms in April – May 2020 during the lockdown in Russia.

**Methods:**

Three hundred and four participants were recruited online (M_age_=33.18, SD=13.33, 108 males, 194 females). Participants completed the Short Schwartz’s Value Survey (SSVS; Lindeman & Verkasalo, 2010). They were next asked to rate how likely their values could be threatened because of the Covid-19. They also completed the State-Trait Anxiety Inventory (Spielberger, 1983) and Beck Depression Inventory-II (Beck et al., 1996).

**Results:**

A multiple linear regression model was built to assess how own values and values threatened by Covid-19 explain state anxiety during the lockdown. Threat to openness values was positively related to state anxiety (b=1.07, SE=.49, β=.13, p=.032). Threat to conservation values was only marginally related to state anxiety (b=1.03, SE=.58, β=.13, p=.074). The effects of self-enhancement and self-transcendence values were not significant.

**Conclusions:**

When Covid-19 is perceived as a threat to openness to change values – hedonism, stimulation and self-direction – people experience higher level of anxiety symptoms. Interestingly, perceived threat of Covid-19 to security, conformity and tradition was only marginally related to anxiety. Future studies might explore how encouraging people to fulfill their openness to change values in a safe mode might decrease the level of anxiety.

